# Comparing post-operative pain between single bundle and double bundle anterior cruciate ligament reconstruction: a retrospective study

**DOI:** 10.1186/s12891-021-04635-5

**Published:** 2021-09-03

**Authors:** Chaiwat Chuaychoosakoon, Wachiraphan Parinyakhup, Arnan Wiwatboworn, Peeranut Purngpiputtrakul, Pawin Wanasitchaiwat, Tanarat Boonriong

**Affiliations:** Department of Orthopedics, Faculty of Medicine, Prince of Songkla University, 15 Karnjanavanich Road, Hat Yai, Songkhla, 90110 Thailand

**Keywords:** Anterior cruciate ligament, Arthroscopic surgery, Post-operative pain, Reconstruction

## Abstract

**Background:**

In anterior cruciate ligament (ACL) reconstruction, the clinical outcome and level of post-operative pain are important factors. To date there have been no studies evaluating differences in post-operative pain between single bundle and double bundle ACL reconstruction with a hamstring graft.

**Hypothesis/purpose:**

We hypothesized that post-operative pain in single bundle ACL reconstruction would be less than in double bundle ACL reconstruction. This study was to compare post-operative pain between patients undergoing single bundle versus double bundle ACL reconstruction.

**Study design:**

Cohort study.

**Methods:**

﻿This was a retrospective study comparing post-operative pain scores between single bundle and double bundle ACL reconstruction. Each patient was given our standard regimen of oral diclofenac (25 mg/tab) three times per day and paracetamol (500 mg/tab) six times per day for 1 day post-operatively. If the patient complained of moderate to severe pain (pain numeric rating scale (PNRS) > 3), 3 mg of morphine was injected intravenously every 3 h for 24 h and 1 mg of morphine as a rescue medication every 1 h for 24 h. PNRS and morphine consumption were recorded at 4-h intervals for 24 h.

**Results:**

209 patients were included in this study of whom 102 and 107 patients received single bundle and double bundle ACL reconstruction, respectively. The average post-operative pain scores of the single bundle group were lower at all time points. Linear mixed effect regression analyses showed that the single bungle group had lower post-operative pain than the double bundle group after adjusting for confounders (beta = − 0.45; 95% CI = − 0.838, − 0.062) but there was no statistically significant difference between numbers of bundle ACL reconstruction with regard to morphine consumption.

**Conclusion:**

Single bundle ACL reconstruction had significantly lower post-operative pain scores than double bundle ACL reconstruction.

**Clinical relevance:**

Double bundle ACL reconstruction results in higher post-operative pain, which may slow the start of rehabilitation and reduce patient satisfaction. In middle-aged adult patients with low-demand activities, we suggest performing a single bundle ACL reconstruction.

## Introduction

The reported pain levels following arthroscopic anterior cruciate ligament (ACL) surgery depend on various factors such as pre-operative pain toleration [1–4], associated intraarticular knee pathologies and operative procedure [2, 5], but regardless of the particulars, such pain is an obstacle to beginning early post-operative rehabilitation and also affects the satisfaction of the patient concerning their operation.

In ACL reconstruction, there are two widely used autograft types, bone-patellar-tendon-bone (BPTB) and semitendinosus-gracilis (STG). Gupta et al. compared the degree of post-operative pain between the BPTB and STG methods in single bundle reconstruction and found that post-operative pain was higher in the BPTB group [6]. As well as discussing the graft choice with the patient in ACL reconstruction, the number of bundles used is also an issue. Normally, either single bundle or double bundle grafts can be used in ACL reconstruction. There are many studies comparing the clinical and functional outcomes (Lysholm scores, Tegner scores, subjective International Knee Documentation Committee (IKDC) between single bundle and double bundle grafts in ACL reconstruction which have reported no significant differences between the two groups [7–9]. Because of these results, differences in the level of post-operative pain between the two methods becomes an important factor in making the decision about which type of ACL reconstruction the patient wishes.

There has one been only one study comparing post-operative pain between the two methods, which reported that double ACL reconstruction resulted in higher post-operative pain than single bundle reconstruction [10]; however, this was a preliminary report presented at a conference via abstract without full details, which have to date not been published. The purpose of this study was to compare post-operative pain between patients undergoing single bundle versus double bundle ACL reconstruction. We hypothesized that post-operative pain in single bundle ACL reconstruction would be less than in double bundle ACL reconstruction.

## Materials and methods

**Fig. 1:**
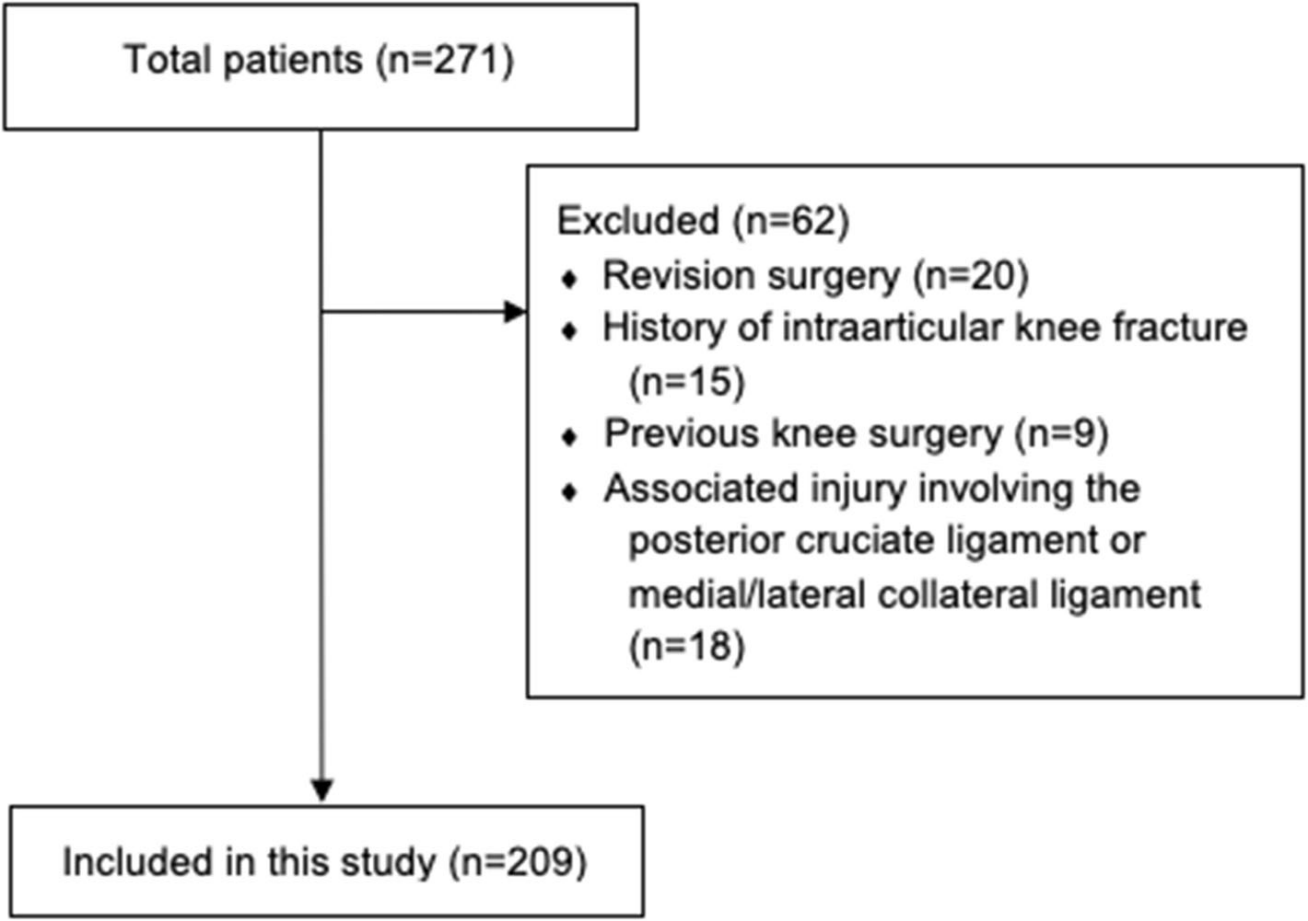
Enrollment flowchart

This study was a retrospective chart review of 271 records of patients aged between 18 and 50 years old who had received arthroscopic ACL reconstruction from January 2016 to December 2019, of which 62 records were excluded. (Fig. 1). Patients who had undergone a revision ACL reconstruction or who had a history of intraarticular knee fracture or previous knee surgery and associated injuries involving the posterior cruciate ligament or medial/lateral collateral ligament were excluded (Fig. 1). The study was approved by the Ethics Committee of the Faculty of Medicine of Prince of Songkla University, which also agreed to waive informed consent. All procedures were carried out in accordance with relevant guidelines and regulations.

Demographic data such as age, gender, body mass index (BMI), side of operation, concomitant intraarticular injuries (meniscal injury and/or cartilage injury), concomitant surgery (meniscal repair), post-operative pain scores and volume of morphine use were recorded with a standard recording form. The choice of single bundle or double bundle ACL reconstruction was made depending on the physician preference. Single bundle ACL reconstruction was performed by T.B., while double bundle ACL reconstruction was performed by W.P.. All patients were followed for 1 day after their surgery to assess their post-operative pain and amount of morphine consumption. Post-operative pain scores and volume of morphine consumption were recorded every 4 h for the first day after the surgery. The post-operative pain was assessed using an 11-point pain numeric rating scale (PNRS), with 0 representing no pain and 10 the worst pain imaginable.

### Surgical procedures

The single bundle ACL reconstructions were performed by a single experienced sports medicine orthopedist (T.B.). Briefly, in each procedure, the patient was placed in the supine position with a thigh tourniquet. An oblique incision was made over the pes anserinus. The semitendinosus and gracilis tendons were harvested and a six-strand bundle prepared for the reconstruction. An anterolateral viewing portal was created, and anteromedial and accessory anteromedial portals for instrumentation. An arthroscopic examination was first done to evaluate the intraarticular pathology. Then, the centers of the femoral and tibial footprints were used as landmarks to create femoral and tibial tunnels, the size depending on the graft size.

The double bundle ACL reconstructions were done by another experienced sports medicine orthopedist (W.P.). The procedures of graft harvesting and portal creation were the same as in the single bundle reconstruction. A 3-strand bundle was prepared from the semitendinosus tendon for the AM bundle and a 3-strand bundle was prepared from the gracilis tendon for the PL bundle. Anteromedial and posterolateral femoral tunnels and two tibial tunnels were created based on the anatomical footprint of the AM and PL bundles.

In all procedures, an EndoButton (Smith & Nephew Endoscopy, Andover, MA) was used for femoral fixation and a Biosure HA (Smith & Nephew Endoscopy, Andover, MA) for tibial fixation.

### Analgesic protocols

#### Intraoperative analgesia

Before the first incision, the patient was anesthetized by an experienced anesthesiologist with a combination spinal nerve block of 3.5% heavy Marcaine with an adductor nerve block using 0.25% Marcaine. The level of the analgesic block was at least L2.

#### Post-operative analgesia

Following the operation, each patient was given oral analgesia of diclofenac (25 mg/tab) three times a day and paracetamol (500 mg/tab) six times per day for 1 day. Both reconstruction groups underwent the same early rehabilitation protocol. All patients were fitted with a locked long knee brace in full extension. Isometric quadriceps exercises and active straight leg raising were started on post-operative day 1. If a patient complained of moderate or severe pain (PNRS > 3), 3 mg of morphine would be given intravenously every 3 h for 24 h and 1 mg of morphine as a rescue analgesic every 1 h.

### Statistical analysis

Continuous data such as post-operative pain scores, cumulative doses of morphine, operative time, and tourniquet time were analyzed using mean ± standard deviation and the paired t-test and ANOVA. Categorical data such as gender, concomitant intraarticular injuries (cartilage injury, meniscal injury), concomitant surgery (meniscal repair) were analyzed with the chi-squared test. We used multivariate linear mix effect regression to assess difference in post-operative pain score between single bundle and double bundle reconstruction techniques with adjustment for confounding by post-operative time, age, sex, concomitant surgery (meniscal repair), and concomitant intraarticular injuries (meniscal or cartilage injuries). A *p*-value of 0.05 was considered significant. Statistical analyses were done using the R program and epicalc package (version 3.4.3; R Foundation for Statistical Computing, Vienna, Austria).

## Results

### Participants

The study included 209 patients, 187 males and 22 females. Single bundle or double bundle ACL reconstructions were performed on 102 and 107 patients, respectively. The demographic data are shown in Table 1, with no statistically significant differences in baseline characteristics between the single bundle and double bundle reconstruction groups except average age and tourniquet time.

**Table 1:** Patient characteristics comparing the single bundle reconstruction and double bundle reconstruction groups

Characteristic	Single bundle (102)	Double bundle (107)	*p*-value
Age (SD)	33.0 (10.5)	29.8 (8.3)	0.015
Body mass index (SD)	24.2 (3.5)	24.6 (4.5)	0.381
Side			0.113
• Right	46 (45.1)	61 (57.0)	
• Left	56 (54.9)	46 (43.0)	
Level of spinal block			0.841
• T10–12	64 (64.0)	66 (61.7)	
• L1-L2	36 (36.0)	41 (38.3)	
Tourniquet time (minutes)	85.0 (39.5)	100.0 (27.0)	< 0.001
Concomitant intraarticular injuries			> 0.05
• Medial meniscal injury	58 (56.9)	65 (60.7)	
• Lateral meniscal injury	47 (46.1)	46 (43.0)	
• Cartilage injury	12 (11.8)	11 (10.3)	
Concomitant surgery			> 0.05
• Medial meniscal repair	31 (30.4)	42 (39.2)	
○ 1 suture	10 (9.8)	12 (11.2)	
○ 2 sutures	15 (14.7)	18 (16.8)	
○ 3 sutures	5 (4.9)	8 (7.5)	
○ 4 sutures	1 (1.0)	4 (3.7)	
• Lateral meniscal repair	17 (16.7)	22 (20.6)	
○ 1 suture	5 (4.9)	7 (6.5)	
○ 2 sutures	9 (8.8)	12 (11.2)	
○ 3 sutures	2 (2.0)	2 (1.9)	
○ 4 sutures	1 (1.0)	1 (1.0)	

Single bundle reconstruction versus Double bundle reconstruction*: PNRS.*

**Fig. 2:**
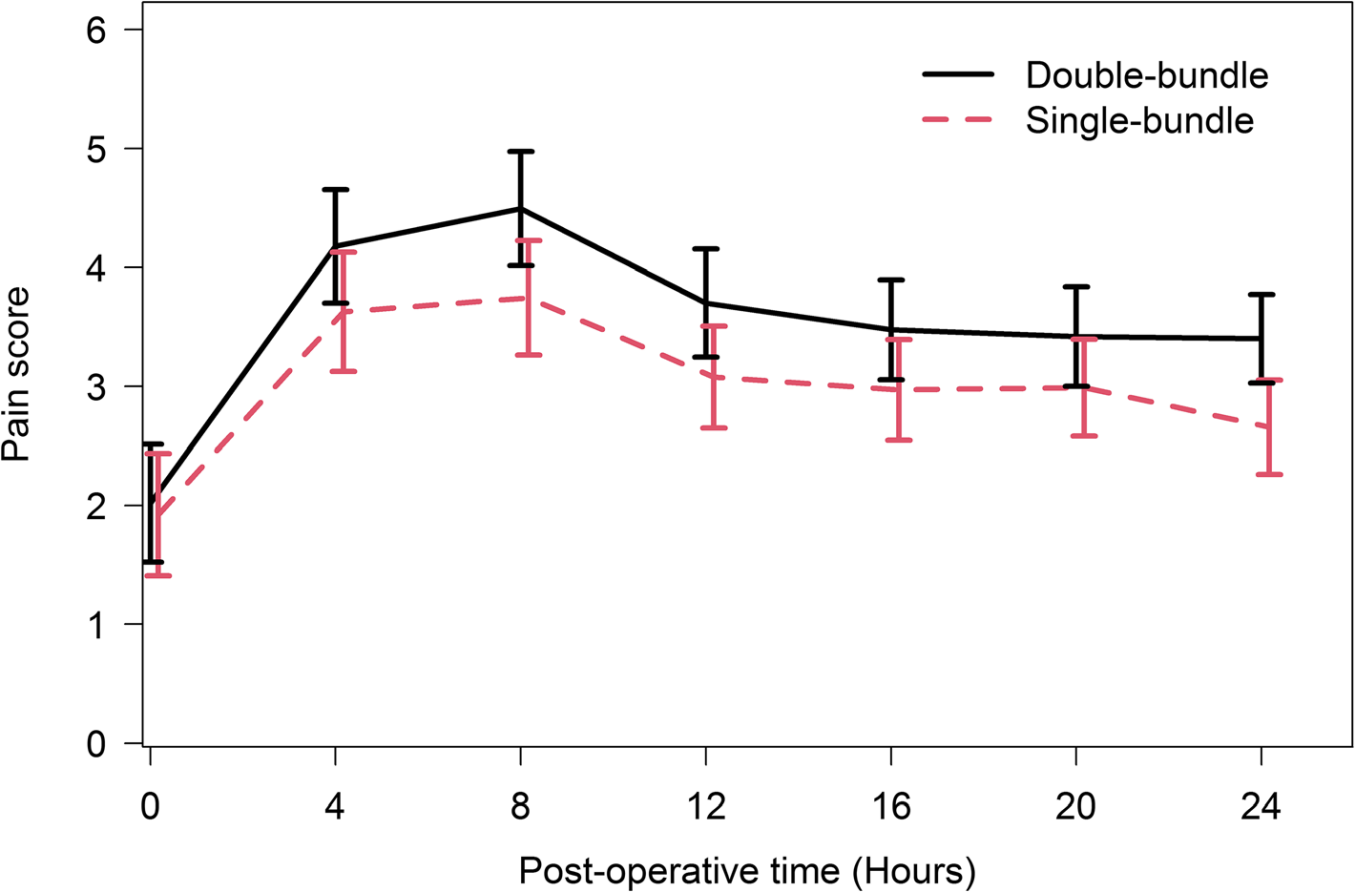
Comparing average post-operative pain scores between the single bundle and double bundle ACL reconstruction groups during the first 24 h after surgery

The average post-operative pain scores (Fig. 2) were lower in the single bundle group than in the double bundle group at all post-operative time points. Linear mixed effect regression analyses (Table 2) showed that the average post-operative pain score in the single bundle group was 0.45 points lower than the score in the double bundle group (beta = − 0.45; 95% CI = − 0.838, − 0.062). The model also showed that for every one-year increased in age, the post-operative pain score decreased by an average of 0.04 point (beta = − 0.04, 95% CI = − 0.060, − 0.020).

**Table 2:** Parameters of the generalized linear model on the association between singe bundle and double bundle ACL reconstruction groups and post-operative pain with potential confounders (*N* = 209 patients) (* Statistically significant, *p*-value < 0.05)

Variable	Regression Coefficient (beta)	Standard Error	95% CI (Lower)	95% CI (Upper)	***P***-value
Numbers of bundle ACL Reconstruction: double bundle (Ref. single bundle)	−0.45	0.198	−0.838	− 0.062	0.0228*
Post-operative time (Ref. 0 h)					
4 h	1.94	0.193	1.562	2.318	< 0.001*
8 h	2.16	0.193	1.782	2.538	< 0.001*
12 h	1.43	0.193	1.052	1.808	< 0.001*
16 h	1.26	0.193	0.882	1.638	< 0.001*
20 h	1.24	0.193	0.862	1.618	< 0.001*
24 h	1.07	0.193	0.692	1.448	< 0.001*
Age in years (continuous)	− 0.04	0.010	− 0.060	− 0.020	< 0.001*
Sex: Female (Ref. Male)	0.52	0.317	− 0.101	1.141	0.106
Concomitant intraarticular injuries (Ref. no injury)	0.04	0.274	−0.497	0.577	0.885
Meniscal repair (Ref. no repair)	−0.16	0.227	−0.605	0.285	0.487

Single bundle reconstruction versus Double bundle reconstruction*: Morphine consumption.*

**Fig. 3:**
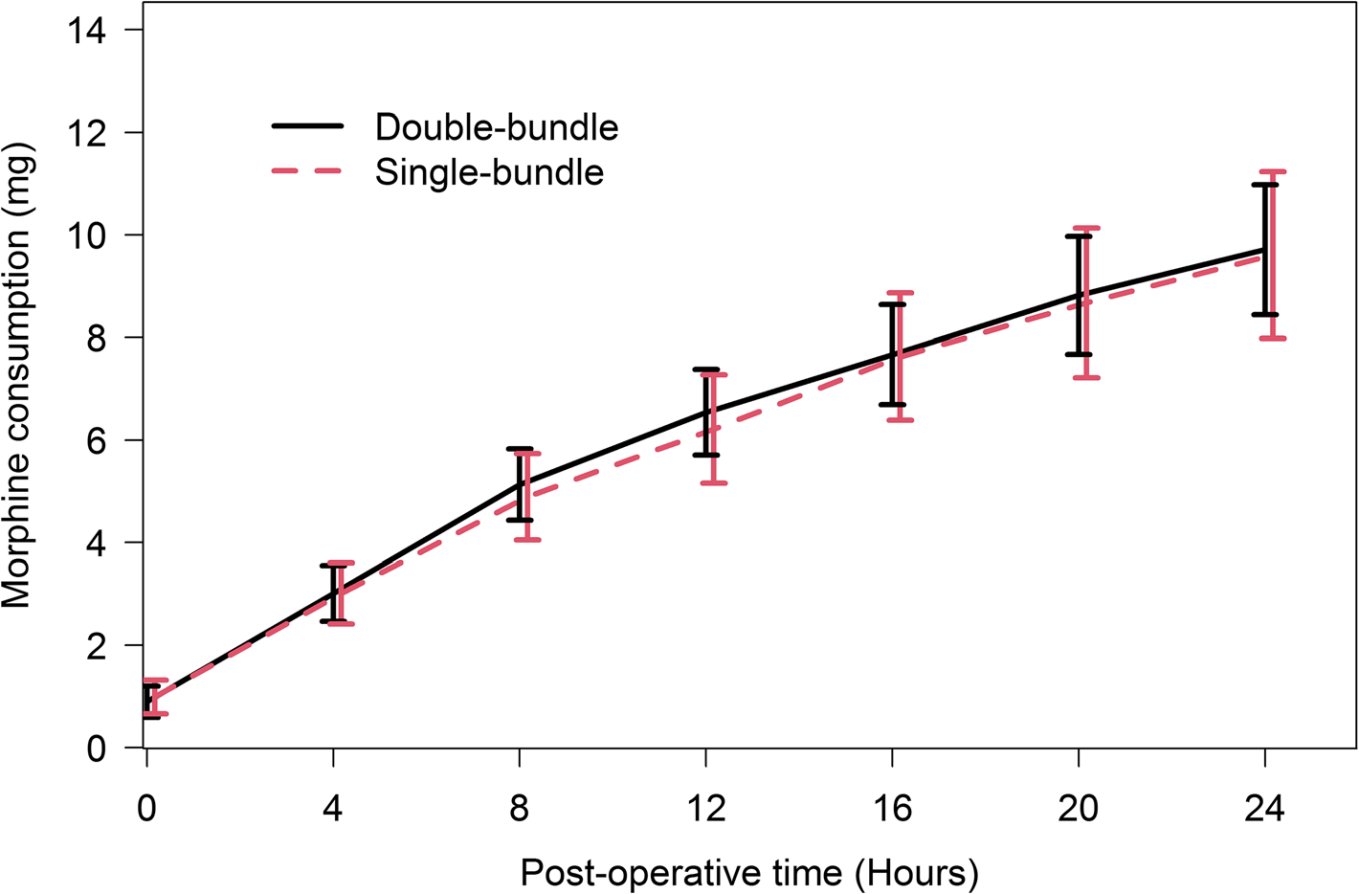
Comparing average cumulative doses of morphine between the single bundle ACL reconstruction and double bundle ACL reconstruction groups during the first 24 h after surgery

The cumulative doses of morphine consumed during the post-operative period in the single bundle and double bundle groups are shown in Fig. 3. There were no statistically significant differences between two groups at all time points (*p*-value ≥0.05).

## Discussion

The single bundle ACL reconstruction group had consistently lower post-operative pain scores than the double bundle ACL reconstruction group, but the cumulative doses of morphine were not different between the two groups.

In recent years, many studies have compared the outcomes between single bundle and double bundle grafts in ACL reconstruction, and overall found no significant differences in clinical and functional outcomes (Lysholm scores [11–20], Tegner scores [12, 14, 17, 19, 21], subjective IKDC [8, 12, 16, 17, 19, 22–24]), although some studies reported that the double bundle ACL reconstruction gave better rotational stability as assessed by the pivot shift test [16, 22, 25] and objective IKDC [16, 21, 26]. The disadvantages of the double bundle ACL reconstruction are that it is technically demanding, has a longer operation time, and it is more difficult to do revision surgery in case of a re-ruptured ACL [27]. Due to the comparatively equal clinical outcomes between single and double bundle ACL reconstructions, differences in the level of post-operative pain between the two methods becomes an important factor in making the decision about which technique to use. Additionally, post-operative pain is one of the most important factors that affect the rehabilitation program and the patient’s satisfaction [6, 28, 29]. Moderate to severe post-operative pain can delay the start of the rehabilitation program thus increasing the chance of a poor functional outcome [6, 28].

A literature review found only one preliminary study comparing post-operative pain between single bundle and double bundle reconstruction, which reported that the double bundle group had higher post-operative pain than the single bundle group [10], similar to our study. Our study found the average post-operative pain scores in the single bundle group were lower than in the double bundle group at all post-operative time points, furthermore linear mixed effect regression analyses showed that the average post-operative pain score was affected by numbers of bundle ACL reconstruction and age. Higher post-operative pain following double bundle reconstruction is probably related to the number and size of the bone tunnels used between the two techniques. Single bundle reconstruction uses one large bone tunnel in both the femur and tibia while double bundle reconstruction uses two small bone tunnels in both bones. While age related post-operative pain in this study was similar to the systematic review of Lautenbacher et al. [30] which reported that middle-aged adult patients had lower pain sensitivity than young adult patients because of higher pain thresholds. In clinical application, we suggest the surgeon should perform a single bundle ACL reconstruction in middle-aged adult patients rather than the double bundle ACL reconstruction because of the levels of post-operative pain scores and generally lower levels of demanding activities in this patient group. In contrast, the surgeon can do a double bundle ACL reconstruction in young adult patients, but the surgeon should be prepared for more intense post-operative pain control.

There were several limitations to this study. First, there are many factors that can affect post-operative pain such as pain toleration, anxiety and psychological stress that were not evaluated in this study. Second, this study was a retrospective study thus there was a risk of selection bias; however, we evaluated the risk of selection bias by analyzing the baseline characteristics of the patients and confirmed there were no statistically significant differences between the single and double bundle reconstruction groups except age and tourniquet time. However, we used multivariate linear mix effect regression to assess difference in post-operative pain score between single bundle and double bundle reconstruction techniques with adjustment for confounding to determine the results. Third, the PNRSs are recorded by the on-duty orthopedic nurses, and we did not attempt to ensure they had all done these assessments in a consistent way; however, the potential for bias was low because post-operative assessments are done following a standard protocol. Fourth, each surgical technique was done by a different orthopaedist, which could have influenced the results by a chance of selection bias. However, we minimized this bias by analyzed the results with regression model. Additionally, both surgeons are well experienced in both procedures which minimizes the learning curve of each procedure; T.B. is more highly experienced in single bundle ACL reconstruction and performed all of these procedures, while W.P. is more highly experienced in double bundle ACL reconstructions and performed all of these procedures.

## Conclusion

Single bundle ACL reconstruction had significantly lower post-operative pain scores than double bundle ACL reconstruction, but the cumulative doses of morphine consumption were not different between the two groups.

## Data Availability

The datasets used and/or analysed during the current study are available from the corresponding author on reasonable request.

## Abbreviations

ACL: Anterior cruciate ligament, BMI: Body mass index
BMI: Body mass index
BPTB: Bone-patellar-tendon-bone, IKDC: International Knee Documentation Committee
IKDC: International Knee Documentation Committee
STG: Semitendinosus-gracilis
PNRS: Pain numeric rating scale
